# Myofascial trigger points in cluster headache patients: a case series

**DOI:** 10.1186/1746-160X-4-32

**Published:** 2008-12-30

**Authors:** Elena P Calandre, Javier Hidalgo, Juan M Garcia-Leiva, Fernando Rico-Villademoros, Antonia Delgado-Rodriguez

**Affiliations:** 1 Institute of Neuroscience, University of Granada, Granada, Spain

## Abstract

Active myofascial trigger points (MTrPs) have been found to contribute to chronic tension-type headache and migraine. The purpose of this case series was to examine if active trigger points (TrPs) provoking cluster-type referred pain could be found in cluster headache patients and, if so, to evaluate the effectiveness of active TrPs anaesthetic injections both in the acute and preventive headache's treatment. Twelve patients, 4 experiencing episodic and 8 chronic cluster headache, were studied. TrPs were found in all of them. Abortive infiltrations could be done in 2 episodic and 4 chronic patients, and preemptive infiltrations could be done in 2 episodic and 5 chronic patients, both kind of interventions being successful in 5 (83.3%) and in 6 (85.7%) of the cases respectively. When combined with prophylactic drug therapy, injections were associated with significant improvement in 7 of the 8 chronic cluster patients. Our data suggest that peripheral sensitization may play a role in cluster headache pathophysiology and that first neuron afferent blockade can be useful in cluster headache management.

## Introduction

Although many advances have been made concerning the pathophysiology of cluster headache, the process is still only partially understood [1]. Hypothalamic dysfunction and subsequent activation of the trigeminovascular system and the cranial parasympathetic system seem to play a significant role [2]. However, other factors may also play a relevant role in the generation and/or perpetuation of cluster pain.

Referred pain arising from myofascial structures has been shown to be involved in many different disorders, mostly of musculoskeletal origin, as it is the case of myofascial pain syndrome [3] but also of visceral origin such as renal calculi, biliar calculi, dysmenorrhea, or myocardial infarction among others [4-6]. Active myofascial trigger points (MTrPs) whose pressure provoked referred pain reproducing the typical patient's headache, have been described both in chronic tension type headache [7] and in migraine [8], indicating that peripheral sensitization may play a role in the pathophysiology of headache. Also Ashkenazi and Young reported the occurrence of cutaneous allodinia in cluster headache, especially prevalent in chronic patients with a longer duration of the disease, a finding which suggests that central sensitization may also be present in this condition [9]. As cluster headache and migraine share some common features concerning trigeminovascular system involvement and response to some abortive and prophylactic drugs, we hypothesized that first neuron hypersensitivity could be also present in patients with cluster headache. Also, just at a clinical level, we noticed that some patients experiencing cluster headache reported that sometimes their attacks were triggered by the wind or when combing their hair, data suggesting the existence of peripheral sensitization.

Based on all the above mentioned data, we decided to examine patients with cluster headache in order to ascertain if referred pain of cluster characteristics suggestive of peripheral sensitization could be observed and, if present, to explore the potential usefulness of anaesthetic injections on pain management. We present herein the results of a case series of patients.

### Case description

We explored patients suffering from cluster headache attending our headache pain unit asking for treatment. Their clinical data were reconsidered to confirm that the diagnosis was in accordance to the International Headache Society revised criteria [10]. Every patient gave informed consent to be examined and, when applicable, injected. In every case an exploration of the scalp and neck was performed by a physician specifically trained in a pain clinic in the detection and manipulation of TrPs, and by means of bilateral finger palpation with a pressure not exceeding 4 kg (i.e. when the pressure applied provoked the typical "blanching of nail" of the explorer's finger). Active trigger points (TrPs) were defined as those areas whose palpation elicited referred pain which reproduced, by its location, characteristics, and/or time sequence, the patient's typical cluster attack. Referred pain of non-cluster characteristics as well as tenderness in the explored areas, if present, were not taken in account for injection since we considered that they did probably not reflected peripheral sensitization due to cluster headache.

In most of the cases, the inactivation of the active myofascial trigger points was done by inserting a needle inside the taut band of the MTrP and injecting 3% mepivacaine; after waiting a couple of minutes the exploration was repeated in order to check if the TrP had been inactivated. When this was not the case, the injection was repeated as necessary up to a maximum of 1 ml of drug en each MTrP, some of them requiring up to 4–5 injections during the same session. However, some muscles – as it is the case of the external pterigoideus -are almost impossible to explore directly due to their location; in this case the exploration was done, according to the well-known diagrams of Simons et al [11], examining the zone of influence this muscle over the skin and subcutaneous surrounding areas and injecting at this level.

Injections were done in the following circumstances: a) when the patient was experiencing a full blown cluster attack in order to see if the injection was able to stop the attack (abortive therapy), b) within one and four hours before the usual time of the attack occurrence in order to ascertain if it could be prevented (preemptive therapy) and c) in a regular basis, one to five days weekly depending on patients' evolution and availability to come to the pain unit (prophylactic therapy). Conditions a) and b) were performed in patients whose attacks followed a fairly regular schedule during the day, appointing them around the time when an attack was expected to occur. Condition c) was applied exclusively to those patients suffering chronic cluster headache; those patients filled a headache diary in order to control the frequency of the cluster attacks along the study period.

Twelve male consecutive patients were studied. Their demographic and clinical characteristics are shown in Table 1; all of the subjects with chronic cluster headache were totally refractory to their current prescribed pharmacological treatment which are also shown in the table; previous failed prophylactic drugs included prednisone, lithium, verapamil, β blockers (propranolol, nadolol), ergotamine, chlorpromazine, amitriptyline, carbamazepine, methysergide, flunarizine, and topiramate. In most of of the cases, the mean duration of the attacks was of two to three hours, although some of them were shorter. None of the patients suffered any other chronic nor severe medical associated condition. Every patient showed at least one active TrP eliciting referred pain of cluster characteristics (Table 2). Thirteen (28.9%) of them were found in temporalis muscle, 8 (17.8%) in external pterygoideus muscle, 5 (11.1%) in suboccipital area, the remaining ones being disseminated in different heterogeneous areas. In 9 (75%) patients the location of active TrPs was consistent with pain laterality; however, in three cases, this correlation failed: two patients suffering right side cluster headache had bilateral active TrPs and one patient with bilateral alternating cluster attacks had active TrPs located exclusively on the left side.

**Table 1:** Demographic and clinical data of patients

Patient	Sex	Age	Diagnosis (location)	Illness duration (years)	Attacks' frequency (daily)	Current prophylactic drugs
1	M	45	episodic (R)	20	1–2	------
2	M	39	episodic (L)	21	1	------
3	M	40	episodic (A)	12	3–4	------
4	M	51	episodic (R)	12	2	------
5	M	43	chronic (A)	13	1–2	verapamil
6	M	48	chronic (A)	21	3–4	valproate
7	M	47	chronic (L)	10	1–4	verapamil + lithium
8	M	36	chronic (A)	16	5–6	verapamil+gabapentin+baclofen
9	M	33	chronic (R)	25	3–4	daily zolmitriptan
10	M	31	chronic (R)	4	4	verapamil+lithium+valproate
11	M	46	chronic (L)	2	2	verapamil
12	M	38	chronic (L)	6	1–2	prednisone

R: right side; L: left side; A: alternating one side and other

**Table 2:** Number of trigger points and results of anaesthetic trigger points injections

Patient	Number and location of TrPs*	Abortive injection	Preemptive injection	Prophylactic injection: mean decrease in attacks' frequency	# of injections/time (months)
1	1: (R) T	N/D	effective	------	3
2	2: (L) P, T	effective	N/D	------	6
3	4: (B) B, EP	effective	effective	------	10
4	2: (R) SO, T	N/D	N/D	------	3
5	4: (B) SO, T	N/D	N/D	50%	24/7
6	6: (B), EP, M, T	N/D	N/D	100%**	12/3
7	9: (L) A, EP, F, P, R, ST, S, T	effective	effective	100%	32/8
8	5: EP, M, P, SD, T	N/D	not effective	no change	24/6
9	3: (L) SD, (B)T	not effective	effective	50%	17/4
10	4: (B) SO, T	N/D	N/D	90%**	24/3
11	1: (L) EP	effective	effective	100%	14/1
12	4: (L) EP, F, N, P, T	effective	effective	90%	14/1

*: A angular, B buccinator, EP external pterygoideus, F frontalis, M masseter, N nasalis, P procerus, R rhomboid, S sternocleidomastoid, SD superior digastric, SO suboccipital group, ST superior trapezius; T temporalis; letters between parenthesis indicate laterality. N/D: not done; **: required a change in the prophylactic drug therapy

As it is shown in Table 2, in 6 patients we could perform an anaesthetic injection just at the beginning of the attack and in 5 (85%) of them the cluster was aborted in a period of time which varied from patient to patient but never exceeded 15 minutes in the most refractory cases. Also, in 7 patients we were able to perform a preemptive injection and in 6 (86%) of them the attack was prevented. The use of anaesthetic injections associated to prophylactic drug treatment in the eight chronic cluster headache patients, was shown to reduce attacks' frequency in at least 50% from their baseline status, in 7 (88%) of them and, in most of the cases, the severity of the attacks, as well as their usual duration, were also decreased. In two patients, the partial improvement observed with needling was substantially increased after changing the prescribed prophylactic drug.

No patient reported relevant side effects related with injections. Pain at injection site was reported by one patient, and rebound headache following injection was reported by four patients.

## Discussion and evaluation

The cases we have presented were not selected from a broader sample, but were the first consecutive cluster patients who we had the opportunity to observe. Unexpectedly, active trigger points were found in every subject examined. It seems, therefore, that peripheral sensitization can be a non infrequent associated feature in cluster headache, at least in those patients whose attacks are not easily controlled.

Myofascial pain is believed to play a relevant role in the pathogenesis of tension-type headache and the presence of active MTrPs related with headache severity and duration has been described in chronic tension-type headache patients [7]. In migraine patients, where pain is essentially related with the activation of the trigeminovascular system, active MTrPs have also been found [8,12] and their inactivation by means of repeated anaesthetic injections has been shown to decrease the frequency and severity of attacks in patients experiencing severe and treatment-refractory migraine [13]. To our knowledge, however, the possibility that active MTrPs could also been found in cluster headache has not been previously investigated. Nevertheless, we believe that the mechanism underlying the presence of active TrPs in each of these primary headaches is similar: chronic pain or repeated acute pain sensitize muscular nociceptors creating active TrPs which, in turn, contribute to potentiate headache pain. This kind of vicious cycle explains why the number of active MTrPs has been found to be higher in patients with chronic primary headaches than in healthy subjects or in patients experiencing less frequent headache attacks [7,8].

Needling therapies are a widely accepted method in myofascial trigger points management, although the basis of its potential effectiveness has not been fully clarified [14,15]. In the treatment of migraine, injection of tender areas during the attack with either lidocaine or saline was shown to subside the pain [16], and the prophylactic use of anaesthetic injections, as mentioned above, was shown to contribute to patients' improvement [13]. In relation to cluster headache, several uncommonly used therapeutic alternatives may, at least in part, act by provoking a first neuron afferent blockade; this would be the case of great occipital nerve blockade, great occipital nerve stimulation, sympathetic C7 anaesthetic blockade, and intranasal lidocaine or cocaine administration [17-21]. Our data, although uncontrolled, suggest that active TrPs injections can be also highly effective in cluster attacks management, either as abortive or as preemptive therapy, and may be of help in chronic cluster management. As it is estimated that between 10–20% of cluster patients are refractory to treatment or develop resistance to it [1], the potential role of active TrP blockade should not be underestimated. Trigger points injection is an easy to perform and well tolerated therapy, its main drawback being that it is rarely wholly effective as monotherapy [22]. The combination of active TrPs inactivation with prophylactic drug treatment in the treatment of refractory cluster headache remains a therapeutic option worthy to be investigated.

## Conclusion

This was just a preliminary and uncontrolled research and, as such, our results should taken with caution. Also, it must be stated that all of the patients attending our pain clinic have been previously diagnosed and treated by other physicians, so that the sample may be not representative of the average cluster headache patient. With these limitations in mind, we think that our data indicate that first neuron hypersensitivity can be present in cluster headache and that active trigger points inactivation can be a useful complementary therapy.

## Competing interests

The authors declare that they have no competing interests.

## Authors' contributions

JH, JMGL and ADR diagnosed and treated patients. EPC and FR participated in the design of the study and drafted the manuscript. All authors read and approved the manuscript's final version.
